# Ambulance Transports from NCAA Division 1 Football Games

**DOI:** 10.1017/S1049023X24000402

**Published:** 2024-06

**Authors:** Aditya C. Shekhar, Abel Alexander, Michael Simms, Mehek Jahan, Anna Haugen, Michelle Lu, Robert Ball, Jeffrey Clement

**Affiliations:** 1. Icahn School of Medicine at Mount Sinai, New York City, New York, USA; 2. Department of Emergency Management, University of Minnesota, Minneapolis, Minnesota, USA; 3. Augsburg University, Minneapolis, Minnesota, USA

**Keywords:** Emergency Medical Services, emergency preparedness, mass-gathering, prehospital care

## Abstract

**Introduction:**

There is significant public health interest towards providing medical care at mass-gathering events. Furthermore, mass gatherings have the potential to have a detrimental impact on the availability of already-limited municipal Emergency Medical Services (EMS) resources. This study presents a cross-sectional descriptive analysis to report broad trends regarding patients who were transported from National Collegiate Athletic Association (NCAA) Division 1 collegiate football games at a major public university in order to better inform emergency preparedness and resource planning for mass gatherings.

**Methods:**

Patient care reports (PCRs) from ambulance transports originating from varsity collegiate football games at the University of Minnesota across six years were examined. Pertinent information was abstracted from each PCR.

**Results:**

Across the six years of data, there were a total of 73 patient transports originating from NCAA collegiate football games: 45.2% (n = 33) were male, and the median age was 22 years. Alcohol-related chief complaints were involved in 50.7% (n = 37) of transports. In total, 31.5% of patients had an initial Glasgow Coma Scale (GCS) of less than 15. The majority (65.8%; n = 48; 0.11 per 10,000 attendees) were transported by Basic Life Support (BLS) ambulances. The remaining patients (34.2%; n = 25; 0.06 per 10,000 attendees) were transported by Advanced Life Support (ALS) ambulances and were more likely to be older, have abnormal vital signs, and have a lower GCS.

**Conclusions:**

This analysis of ambulance transports from NCAA Division 1 collegiate football games emphasizes the prevalence of alcohol-related chief complaints, but also underscores the likelihood of more life-threatening conditions at mass gatherings. These results and additional research will help inform emergency preparedness at mass-gathering events.

## Introduction

Almost forty million people attend National Collegiate Athletic Association (NCAA; Indianapolis, Indiana USA) Division I collegiate football games in the United States every year, and they often take place in stadiums capable of accommodating tens of thousands of fans at a single event.^1,2^ Ample research has looked at medical care at mass-gathering events. For instance, a review by Milsten, et al^3^ identified several important variables associated with mass gatherings, including weather, event type, attendance, and crowd dynamics (eg, age and alcohol/drug use). Similarly, several studies indicate a relationship between crowd size and number of patients encountered at mass-gathering events, as well as an association between event type and number of patients.^4,5^

However, different types of gatherings attract different types of crowds and are associated with different behaviors, so there is interest in evaluating specific gatherings to inform emergency planning. Relatively little research has examined emergency medical response specifically at collegiate football games or the kinds of medical emergencies encountered at these events. Looking at other types of events can establish a baseline for comparison. For example, data from five different large stadiums indicate medical emergencies occurred roughly between 0.1 and 0.3 times per 10,000 people.^6^ Another study examining cardiac emergencies across 20 different soccer stadiums within the Netherlands found an overall incidence rate of 7.3 per one million spectators.^7^

One challenge with providing medical care at collegiate football games is the presence of alcohol and patients with alcohol-related emergencies. According to a survey by Glassman, et al,^5^ 36% of college students reported drinking more than four or five drinks, and 16% reported drinking more than eight or ten drinks. Spaite, et al^8^ found that a stadium disallowing fans to bring their own alcohol inside did not change the overall incidence of medical emergencies but affected what kinds of illnesses/injuries were encountered. More recently, Ruehlmann, et al^9^ found on-site alcohol sales at a college stadium did not cause a statistically-significant change in Emergency Medical Services (EMS) activations or emergency department visits for alcohol-related chief complaints. The authors postulated that patients may have consumed less alcohol at tailgates or parties before the game because of alcohol being sold on site.^9^ Moreover, college football games have been associated with increased arrests and crime.^10,11^ These findings make clear the importance of emergency preparedness at mass gatherings.

The emergency planning process is aided by examining the incidence rates and chief complaints of patients at a specific class of mass gatherings, and in turn, what resources are required to transport these patients. Records of patients who were transported from collegiate varsity football games at a major public university competing at the highest level of collegiate athletics were examined, providing a descriptive analysis of the kinds of medical emergencies that take place at collegiate athletic events, which in turn informs EMS resource allocation and emergency preparedness efforts.

## Methods

The University of Minnesota-Twin Cities is a large public university in Minneapolis and St. Paul, Minnesota (USA). Their varsity football team competes at the NCAA Division 1 level and participates as part of the Big 10 Conference. Home games are held at the Huntington Bank Stadium (formerly TCF Bank Stadium) on the Minneapolis campus, which has a maximum capacity of 50,805. Dedicated on-site EMS coverage for spectators and staff is provided by University of Minnesota Emergency Medical Services (UMEMS; Minneapolis, Minnesota USA), which provides stand-by Basic Life Support (BLS) response and treatment within the stadium as well as ambulance transport to nearby hospitals. Advanced Life Support (ALS) transport is handled by Hennepin EMS, which is an EMS agency affiliated with Hennepin Healthcare – a local health system in Minneapolis. Pertinent information about patients encountered at Huntington Bank Stadium are recorded on patient care reports (PCRs) by members of UMEMS staff.

In this study, UMEMS PCRs during six years’ worth of football seasons (2015, 2016, 2017, 2019, 2021, and 2022) were examined and a retrospective chart review was conducted.^12^ Trained research assistants (all of whom are experienced EMS clinicians and are familiar with writing PCRs) were employed as abstractors. They manually examined each paper PCR on file to determine if it met inclusion criteria (ie, if the patient required ambulance transportation from the stadium or the immediate vicinity and occurred on the date of scheduled football games during gametime hours). Specific de-identified information was then abstracted from each PCR and compiled using a standardized data collection form.

Variables collected included: age/sex, EMS disposition, chief complaint, whether the complaint was alcohol-related, treatments and medications provided, initial Glascow Coma Scale (GCS) score, and any vital signs dramatically outside normal limits (defined as: systolic blood pressure [SBP] ≥ 160 mmHg, SBP ≤ 90 mmHg, heart rate [HR] ≥ 140 BPM, HR ≤ 50 BPM, blood glucose level [BGL] ≥ 200 mg/dL, BGL ≤ 60 mg/dL, and oxygen saturation/SpO2 ≤ 93%). These data were then analyzed to identify trends in chief complaints and resource requirements. Given expected variation in documentation, different chief complaints, and the sample size, abstraction and analysis focused on these comparatively objective measures to minimize potential bias. Prevalence data were calculated using standardized attendance statistics reported by the University of Minnesota Athletics Department. This study was determined to be exempt from institutional review board (IRB) review by the University of Minnesota IRB (STUDY00016094).

## Results

A total of 73 patient transports resulted from the 4.49 million attendees at 90 games over the course of the analysis: 65.8% (n = 48; 0.11 per 10,000 attendees) were transported by BLS ambulances, while the remaining patients (34.2%; n = 25; 0.06 per 10,000 attendees) were transported by ALS ambulances. The average game had a reported attendance of 49,933, ranging from 21,220 to 111,090. The average number of transports per game was less than one for both ALS (0.3 transports per game) and BLS (0.8 transports per game).

Of these patients, 45.2% (n = 33) were male, and the median age was 22 years (Table 1). Patients between the ages of 18 and 22 accounted for 45.2% of transports (n = 33), and just under 10% (n = 7) of transports involved patients 18 years or younger. Alcohol use or intoxication was related to the chief complaint in 50.7% (n = 37) of transports, and falls were the primary mechanism of injury in 26.0% (n = 19) of transports. Alcohol-related chief complaints involving patients under the legal drinking age (21 years) accounted for 20.5% (n = 15) of transports. Just under one-third of patients presented to EMS with an initial GCS of less than 15 (31.5%; n = 23), and just under ten percent (8.2%; n = 6) had a GCS of less than 10. Patients with alcohol-related chief complaints had a lower average GCS than patients with non-alcohol-related chief complaints (13.2 versus 14.5). Oxygen was provided on-scene to patients in 19.2% of encounters – the median flow rate was five liters per minute. Thirteen transports (17.8%) involved patients with significantly abnormal vital signs on initial EMS encounter.

**Table 1.** Summary Statistics and Transport Breakdown

Variable	Overall N = 73	ALS N = 25	BLS N = 48	P Value
**Age** (mean, SD)	36 (SD = 21)	44 (SD = 25)	32 (SD = 18)	.081*^a^*
Unknown	2	1	1	
**Sex** (n, %)				.6*^b^*
Female	39 (54%)	14 (58%)	25 (52%)	
Male	33 (46%)	10 (42%)	23 (48%)	
Unknown	1	1	0	
**Initial GCS** (mean, SD)	13.82 (SD = 2.25)	13.38 (SD = 2.95)	14.04 (SD = 1.81)	.6*^a^*
Unknown	1	1	0	
**ETOH** (n, %)				.7*^c^*
No	35 (48%)	14 (56%)	21 (44%)	
Yes	28 (38%)	8 (32%)	20 (42%)	
Yes, w/ Injury	10 (14%)	3 (12%)	7 (15%)	

Note: Key data stratified based by Advanced Life Support (ALS) versus Basic Life Support (BLS) Transport.

Abbreviations: ALS, Advanced Life Support; BLS, Basic Life Support; GCS, Glasgow Coma Scale; ETOH, ethanol.

a Wilcoxon rank sum test.

b Pearson’s Chi-squared test.

c Fisher’s exact test.

**Figure 1.**
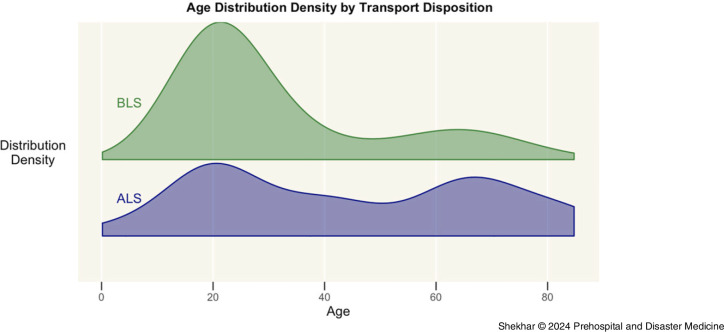
Age Distribution Density by Transport Disposition.

A BLS ambulance was used to transport 65.8% of patients (n = 48), and an ALS ambulance was used to transport just over one-third of patients (34.2%; n = 25). The BLS ambulances were more often used to transport patients with alcohol-related chief complaints: one out of every 1.8 BLS transports involved alcohol-related chief complaints, and one out of every 2.3 ALS transports involved alcohol-related chief complaints. Furthermore, patients transported by ALS ambulance had lower average GCS scores (13.4 versus 14.0; not statistically significant), were more likely to have abnormal vital signs (1/2.8 for ALS versus 1/12 for BLS), and tended to be older (44.3 years versus 31.5 years; *P* = .08) than patients transported by BLS ambulance (Figure 1).

## Discussion

Analysis of patients transported from NCAA Division 1 football games reveals several noteworthy trends. First, nearly one-half of patients transported were college-aged – defined as between 18 and 22 years old. Second, alcohol was related to the chief complaint in just over fifty percent of transports. Focusing on alcohol-related chief complaints reveals that one-fifth of transports involved alcohol-related chief complaints in patients under the legal drinking age. Furthermore, patients with alcohol-related chief complaints had a lower average GCS but were more likely to be transported by BLS ambulance. However, this difference in average GCS is primarily attributable to a handful of patients with low GCS values who were transported by ALS ambulance for airway protection. This echoes earlier studies showing increased alcohol consumption during collegiate football game days.^10,11,13^ Third, there were differences across patients who were transported via ALS ambulance and BLS ambulance. Specifically, patients transported by ALS ambulance had lower average GCS scores, were more likely to have abnormal vital signs, and were more likely to be older than patients transported by BLS ambulance. As mentioned previously, ALS ambulances were often called for patients requiring advanced-level interventions, such as compromised airways.

In reading the PCRs included in this study, it also became apparent that individual on-site providers may have had different ALS/BLS preferences. For instance, one provider may request an ALS ambulance for a patient, whereas a different provider would have instead opted to request a BLS ambulance. Another possibility is that an ALS ambulance may have been requested for some patients, but resource availability at that time meant it would be quicker to transport the patient via BLS ambulance. The stadium in this study is located within minutes from several major hospitals, including a Level 1 trauma center; a BLS ambulance standing by at the stadium can often transport the patient to definitive care at one of these hospitals faster than an ALS ambulance can reach the patient. Furthermore, EMS planning at this stadium has BLS ambulances standing by during games. Thus, the proximity of the stadium to receiving facilities and the availability of BLS ambulances may have influenced transport decisions – perhaps, a greater number of patients would have been transported via ALS ambulance if the stadium was located further away from receiving facilities with the ability to provide comprehensive care. It is likely that BLS ambulances being able to transport patients from the stadium offered relief to potentially-strained ALS ambulances. This may be of interest to EMS services who struggle to staff sufficient ALS resources. Furthermore, the question of BLS ambulances’ utility in mass-gathering emergency medical care requires more research.

## Limitations

This study has several limitations. First, it only considers data from a single stadium in a single city. It is possible that other stadiums or different geographies may be associated with different injury and illness patterns. Second, only six years’ worth of data were available, generating a sample size of only 73 transports. Third, hospital records of patients that were transported are not available to be linked, so the ultimate in-hospital disposition of patients (eg, admitted versus not admitted) is not known. Having said that, this study offers important insight into the kinds of medical emergencies encountered at collegiate football games.

## Conclusion

This study sought to report key information about patients who were transported by ambulance from NCAA Division 1 football games with the hope of informing future emergency preparedness efforts and better defining resource requirements for mass gatherings. The analysis of patients transported by ambulance from NCAA Division 1 collegiate football games reveals several noteworthy findings. These include: (1) the prevalence of alcohol-related chief complaints; (2) differences between patients transported by ALS ambulance versus BLS ambulance; and (3) the rates of abnormal vital signs or GCS scores below 15. These results work and further research will hopefully help inform emergency preparedness at future collegiate football games and other mass-gathering events.
